# The epidemiology and clinical outcomes of ventilator-associated events among 20,769 mechanically ventilated patients at intensive care units: an observational study

**DOI:** 10.1186/s13054-021-03484-x

**Published:** 2021-02-02

**Authors:** Qiao He, Wen Wang, Shichao Zhu, Mingqi Wang, Yan Kang, Rui Zhang, Kang Zou, Zhiyong Zong, Xin Sun

**Affiliations:** 1 Chinese Evidence-Based Medicine Center and CREAT Group, West China Hospital of Sichuan University, Chengdu, 610041 China; 2 Department of Infection Control, West China Hospital of Sichuan University, Chengdu, 610041 China; 3 Intensive Care Unit, West China Hospital of Sichuan University, Chengdu, 610041 China; 4 Information Center, West China Hospital of Sichuan University, Chengdu, 610041 China; 5 Center of Infection Diseases, West China Hospital of Sichuan University, Chengdu, 610041 China

**Keywords:** Ventilator-associated events, Epidemiology, Intensive care units, Ventilator-associated condition, Infection-related ventilator-associated complications, Ventilator-associated pneumonia

## Abstract

**Background:**

Ventilator-associated pneumonia (VAP) is the most common hospital-acquired infection (HAI) in intensive care units (ICUs). Ventilator-associated event (VAE), a more objective definition, has replaced traditional VAP surveillance and is now widely used in the USA. However, the adoption outside the USA is limited. This study aims to describe the epidemiology and clinical outcomes of VAEs in China, based on a prospectively maintained registry.

**Methods:**

An observational study was conducted using an ICU-HAI registry in west China. Patients that were admitted to ICUs and underwent mechanical ventilation (MV) between April 1, 2015, and December 31, 2018, were included. The characteristics and outcomes were compared between patients with and without VAEs. The rates of all VAEs dependent on different ICUs were calculated, and the pathogen distribution of patients with possible VAP (PVAP) was described.

**Results:**

A total of 20,769 ICU patients received MV, accounting for 21,723 episodes of mechanical ventilators and 112,697 ventilator-days. In all, we identified 1882 episodes of ventilator-associated condition (VAC) events (16.7 per 1000 ventilator-days), 721 episodes of infection-related ventilator-associated complications (IVAC) events (6.4 per 1000 ventilator-days), and 185 episodes of PVAP events (1.64 per 1000 ventilator-days). The rates of VAC varied across ICUs with the highest incidence in surgical ICUs (23.72 per 1000 ventilator-days). The median time from the start of ventilation to the onset of the first VAC, IVAC, and PVAP was 5 (3–8), 5 (3–9), and 6 (4–13) days, respectively. The median length of hospital stays was 28.00 (17.00–43.00), 30.00 (19.00–44.00), and 30.00 (21.00–46.00) days for the three VAE tiers, which were all longer than that of patients without VAEs (16.00 [12.00–23.00]). The hospital mortality among patients with VAEs was more than three times of those with non-VAEs.

**Conclusions:**

VAE was common in ICU patients with ≥ 4 ventilator days. All tiers of VAEs were highly correlated with poor clinical outcomes, including longer ICU and hospital stays and increased risk of mortality. These findings highlight the importance of VAE surveillance and the development of new strategies to prevent VAEs.

## Background

Most critically ill patients admitted to intensive care units (ICUs) require life-saving mechanical ventilation (MV), despite the multiple complications associated with it [1–3]. Ventilator-associated pneumonia (VAP) is one such complication and is the most common hospital-acquired infection (HAI) in ICUs [4, 5]. It has been reported that VAP is associated with longer duration of MV, prolonged hospital and ICU stays, long-term disability, higher mortality, and increased hospital costs [1, 2, 4, 6, 7]. Therefore, the surveillance of VAP for the incidence estimates is particularly important for understanding the epidemiology and risk management of VAP.

Traditional definitions of VAP, however, were subjective, complex, and poor both for sensitivity and specificity, which made it difficult to implement [3, 8–13]. In January 2013, a new approach to VAP surveillance—ventilator-associated events (VAEs)—was proposed by a working group convened by the Centers for Disease Control and Prevention (CDC) and comprised members of several stakeholder organizations [3]. The following three definition tiers are embedded within the VAE algorithm: (1) ventilator-associated condition (VAC), (2) infection-related ventilator-associated complication (IVAC), and (3) possible VAP (PVAP). In contrast to traditional VAP, VAE surveillance definitions were objective, streamlined, and potentially automatable that can identify a broad range of conditions and complications, more than VAP, only occurring in mechanically ventilated adult patients [8, 13–15].

Although it has been more than five years since the VAE surveillance was proposed and has replaced the traditional VAP surveillance, its adoption outside the USA seems limited [16]. Several published studies have reported the incidence of VAE. However, the rates varied markedly, ranging from 3 to 77% (6–107 per 1000 ventilator-days) [1, 2, 4, 7, 17–36]. Explanations for the discrepancy may be various study settings and different patient populations used as the denominator for the calculation of VAE rate. These studies were mainly from the USA, followed by Europe, while the population used as the denominator included all MV patients or patients with at least a certain number of ventilator-days. Besides, the majority of these studies were retrospective, in that the completeness and accuracy of data had a great impact on case identification. In China, the surveillance was mainly limited to traditional VAP. Studies on VAE incidence are scarce [7, 21], which may further limit the understanding of comprehensive complications among mechanically ventilated patients in China.

In 2015, a routinely active monitoring module for VAE was established and embedded in an existing ICU-HAI system (an HAI monitoring system in ICU units) in West China Hospital (WCH), China. To our knowledge, this is a unique system carrying out routine surveillance for VAE in China. By linking the HAI system with the electronic medical record (EMR) and ICU systems, we have developed an ICU-HAI registry. We undertook an observational study based on this registry with the aim to evaluate the following: (1) the clinical characteristics and outcomes between patients with and without VAEs; (2) the incident rates of different VAE tiers; and (3) the pathogen distribution in patients with PVAP.

## Methods

This study was approved by the Ethical Committee of West China Hospital in 2018 (WCH2018-409), and the need for patient consent was waived.

### Data sources

We carried out an observational study using an established ICU-HAI registry. A detailed description of this registry has been published elsewhere [37]. In brief, this registry consisted of three databases including an EMR system, an ICU system, and an ICU-HAI monitoring system. The ICU-HAI system was a prospective surveillance system, actively collecting ICU-HAI–related information of all patients admitted to an ICU by a team of three infection control practitioners. Annually, there were more than 8000 person-times for ICU-HAI and 5000 cases for VAE were monitored. The EMR system was established in 2008 and stored patient-level health care and medical information. The ICU system was electronically recorded by well-trained special nurses and contained critical care information regarding vital signs, life support, nurse notes, risk assessment, and ICU-HAI-related checklists.

These three databases were linked via a unique patient identification code, and the linkage rate proved to be 100%. Until December 31, 2018, approximately 30,000 patients were admitted to one of six ICUs [general ICU (GICU), surgical ICU (SICU), neurological ICU (NICU), respiratory ICU (RICU), thoracic surgery ICU (TICU), and pediatric ICU (PICU)]. This registry has been validated and shown to be of high quality.

### Study population and case definition

In this study, patients admitted to either of five ICUs between April 1, 2015, and December 31, 2018, and had at least one day on MV were included. We excluded patients who met any of the following criteria: (1) age < 18 years or admitted to the PICU; (2) incomplete information including date of birth, sex, and discharged diagnosis; (3) extremely long ICU stay and abnormal bill; and (4) non-Chinese nationality.

VAE cases were extracted from the active surveillance module for VAE in the ICU-HAI system. VAE algorithm according to the definition of the CDC’s National Healthcare Safety Network (CDC-NHSN) [3] was implemented into this module, and this module could automatically record the positive end-expiratory pressure (PEEP) and the fraction of inspired oxygen (FiO2) hourly from ventilator parameters, and automatically screen for suspected VAE cases according to this algorithm. Three infection control practitioners judged the type of VAE for each suspected case. The accuracy of PVAP was previously validated to be 96.2% [37]. In this study, we defined: (1) VAC-plus (all patients with VAC, including those who also fulfilled criteria for IVAC and PVAP); (2) IVAC-plus (all patients who met the IVAC criteria, including those with PVAP); and (3) PVAP. An episode of VAE that occurred after the NHSN 14-day repeat infection timeframe of the previous event was defined as a new episode and was included. We defined two groups for non-VAEs: (1) all patients on MV but without any VAE and (2) patients with at least four consecutive ventilator-days but no VAE, as VAE must have four consecutive ventilator-days.

### Data collection

Patient characteristics were extracted, including demographic characteristics (age, sex); ICU type; chronic comorbidities (hypertension, diabetes, cardiovascular disease, malignant tumor, chronic lung disease, liver failure, renal failure, and heart failure); acute comorbidities at ICU admission [gastrointestinal bleeding, shock, pneumonia, and acute respiratory distress syndrome (ARDS)]; APACHE II score; surgery (cardiac or cranial surgery); intubation sites; tracheostomy; time of admission and discharge; time on MV; time of VAE occurrence; and VAE type. The chronic comorbidities were identified through the International Classification of Diseases, 10th edition (ICD-10), and the completeness and accuracy were 99% and 88%, respectively [37]. For acute comorbidities presented at ICU admission, which were stored in transferred summary as unstructured formats, test mining was used to identify related information. Furthermore, the processes of care (head-of-bed elevation, oral care, paired spontaneous awakening trials and breathing trials, stress ulcer prophylaxis, thromboembolism prophylaxis) were also collected.

### Statistics

Descriptive statistics was used to summarize the characteristics and outcomes (length of hospital stay (LOS), length of stay in ICU, length of stay on MV, and hospital and ICU mortality) of non-VAE groups, VAC-plus group, IVAC-plus group, and PVAP group. Continuous variables are presented as median [interquartile range (IQR)] and categorical variables as frequency (percentages). The number of per 100 episodes of mechanical ventilation (EMV) and the number of per 1000 ventilator-days for VAC-plus, IVAC-plus, and PVAP were calculated and stratified by ICU type, discharge date, and time of event onset, respectively. The compliance rates of processes of care were calculated as well. Finally, we listed the pathogen distribution in patients with PVAP.

## Results

### Study population

**Fig. 1:**
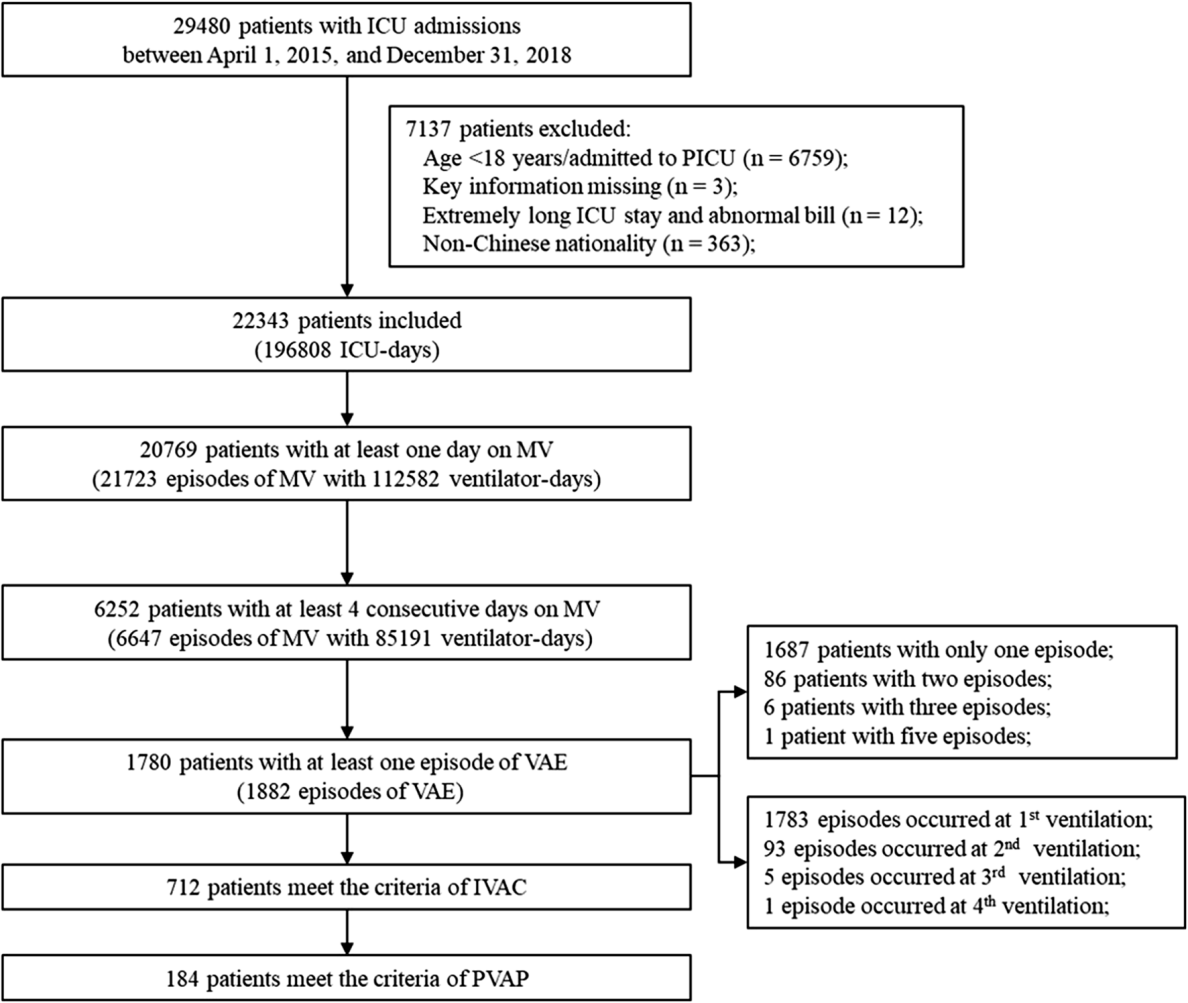
Flowchart of included patients

A total of 22,343 patients admitted to the five ICUs, corresponding to 196,808 ICU-days during the study period were identified. Of these, 20,769 (93.0%) received MV with 21,723 EMV and 112,582 ventilator-days. The median (IQR) time of total ventilator-days was 2 (2–5) days. However, only 6252 patients (28.0% of all ICU patients and 30.1% of those on MV) received MV for at least four consecutive days, while there were 6647 EMV and 86,025 ventilator-days, with a median of 9 (6–16) days (Fig. 1).

### The rates of VAEs

Among the 6252 patients, 1780 (28.5%) experienced at least one episode of VAC-plus, 712 (11.4%) of IVAC-plus, and 184 (2.9%) of PVAP. Ninety-five (1.5%) patients experienced more than one episode and most events occurred at their first ventilation. Among 90 patients with failed extubation attempts, 13 patients further developed a VAE. Table 1 shows the rates of the three VAE tiers. In total, we identified 1882 episodes of VAC-plus events (8.66 per 100 EMV and 16.7 per 1000 ventilator-days), 721 episodes of IVAC-plus events (3.32 per 100 EMV and 6.4 per 1000 ventilator-days), and 185 episodes of PVAP events (0.85 per 100 EMV and 1.64 per 1000 ventilator-days). The rates varied in ICUs (Table 2) from 7.29 (TICU) to 23.72 (SICU) per 1000 ventilator-days for VAC-plus, from 3.59 (TICU) to 9.44 (NICU) per 1000 ventilator-days for IVAC-plus, and from 0.62 (SICU) to 2.18 (NICU) per 1000 ventilator-days for PVAP. However, the rates remained stable with the discharge date. Most VAEs occurred early in the course of the mechanical episode. A total of 1285 (68.3%) VAEs occurred in the first week since ventilation was initiated, with a mean rate of 5.92 per 100 EMV (18.65 per 1000 ventilator-days), and 1616 (85.9%) VAEs occurred within 14 days with 1.52 per 100 EMV (3.77 per 1000 ventilator-days). The mean rate dropped to less than one per 1000 ventilator-days after 21 days. The proportion of IVAC-plus to VAC-plus was 0.38 and ranged from approximately one-third to one-half throughout the whole course of the mechanical episode and in different ICU units. The proportion of PVAP to VAC-plus was 0.1 and was highest in the TICU (0.26).

**Table 1:** The rates of ventilator-associated events by ICU units, discharge date and days from the start of ventilation

	EMV	Ventilator-days	VAC-plus			IVAC-plus			PVAP-plus			Proportion of IVACs: VACs	Proportion of PVAPs: VACs
			Event episodes	Per 100 EMV	Per 1000 ventilator-days	Event episodes	Per 100 EMV	Per 1000 ventilator-days	Event episodes	Per 100 EMV	Per 1000 ventilator-days		
Overall	21,723	112,679	1882	8.66	16.7	721	3.32	6.4	185	0.85	1.64	0.38	0.1
Type of ICU													
GICU	5050	38,638	656	12.99	16.98	243	4.81	6.29	75	1.49	1.94	0.37	0.11
NICU	2291	18,322	392	17.11	21.4	173	7.55	9.44	40	1.75	2.18	0.44	0.1
RICU	1492	16,081	200	13.4	12.44	77	5.16	4.79	22	1.47	1.37	0.39	0.11
SICU	5288	20,994	498	9.42	23.72	161	3.04	7.67	13	0.25	0.62	0.32	0.03
TICU	7602	18,644	136	1.79	7.29	67	0.88	3.59	35	0.46	1.88	0.49	0.26
Discharge date													
2015.4–2016.3	5924	29,123	480	8.1	16.48	197	3.33	6.76	49	0.83	1.68	0.41	0.1
2016.4–2017.3	5776	29,083	494	8.55	16.99	190	3.29	6.53	43	0.74	1.48	0.38	0.09
2017.4–2018.3	5694	30,695	490	8.61	15.96	179	3.14	5.83	51	0.9	1.66	0.37	0.1
2018.4–2018.12	4329	23,778	418	9.66	17.58	155	3.58	6.52	42	0.97	1.77	0.37	0.1
The days from the start of ventilation													
3–7	21,723	68,910	1285	5.92	18.65	474	2.18	6.88	104	0.48	1.51	0.37	0.08
8–14	21,723	87,882	331	1.52	3.77	138	0.64	1.57	40	0.18	0.46	0.42	0.12
15–21	21,723	96,986	114	0.52	1.18	50	0.23	0.52	23	0.11	0.24	0.44	0.2
22–28	21,723	101,801	51	0.23	0.5	21	0.1	0.21	8	0.04	0.08	0.41	0.16
> 28	21,723	112,679	51	0.44	0.84	34	0.16	0.3	8	0.04	0.08	0.36	0.06

*ICU* intensive care units, *EMV* episodes of mechanical ventilation, *GICU* general intensive care units, *SICU* surgical intensive care units, *NICU* neurological intensive care units, *RICU* respiratory intensive care units, *TICU* thoracic surgery intensive care units, *VAC* ventilator-associated conditions, *IVAC* infection-related ventilator-associated complication, *PVAP* possible ventilator-associated pneumonia

**Table 2:** The characteristics and clinical outcomes of patients with and without ventilator-associated events

	Non-VAE with at least one ventilator-day (N = 18,989)	Non-VAE with at least four ventilator-days (N = 4472)	VAC-plus (N = 1780)	IVAC-plus (N = 712)	PVAP (N = 184)
Age, median (IQR)	55.00 (46.00, 65.00)	59.00 (46.00, 70.00)	57.50 (46.00, 69.00)	57.00 (46.00, 69.00)	59.00 (46.00, 70.25)
18–44	4251 (22.4)	950 (21.2)	399 (22.4)	163 (22.9)	40 (21.7)
45–64	9466 (49.9)	1814 (40.6)	726 (40.8)	285 (40.0)	74 (40.2)
65–74	3470 (18.3)	924 (20.7)	367 (20.6)	164 (23.0)	43 (23.4)
≥ 75	1801 (9.5)	784 (17.5)	288 (16.2)	100 (14.0)	27 (14.7)
Female, *n* (%)	7986 (42.1)	1666 (37.3)	639 (35.9)	243 (34.1)	53 (28.8)
Type of ICU, *n* (%)					
GICU	4235 (22.3)	1464 (32.7)	640 (36)	253 (35.5)	82 (44.6)
NICU	1813 (9.5)	841 (18.8)	365 (20.5)	173 (24.3)	41 (22.3)
RICU	1193 (6.3)	850 (19)	196 (11)	80 (11.2)	22 (12)
SICU	4540 (23.9)	743 (16.6)	484 (27.2)	164 (23)	15 (8.2)
TICU	7335 (38.6)	613 (13.7)	141 (7.9)	70 (9.8)	36 (19.6)
Comorbidities, *n* (%)					
Hypertension	3849 (20.3)	982 (22.0)	364 (20.4)	145 (20.4)	33 (17.9)
Diabetes	992 (5.2)	263 (5.9)	78 (4.4)	37 (5.2)	7 (3.8)
Ischemic heart diseases	190 (1.0)	28 (0.6)	12 (0.7)	7 (1.0)	6 (3.3)
Chronic lung diseases	509 (2.7)	272 (6.1)	89 (5.0)	30 (4.2)	11 (6.0)
Pulmonary vasculature diseases	847 (4.5)	276 (6.2)	87 (4.9)	31 (4.4)	14 (7.6)
Cancer	3522 (18.5)	385 (8.6)	137 (7.7)	48 (6.7)	14 (7.6)
Heart failure	4250 (22.4)	475 (10.6)	140 (7.9)	60 (8.4)	20 (10.9)
Liver failure	137 (0.7)	81 (1.8)	49 (2.8)	22 (3.1)	3 (1.6)
Kidney failure	485 (2.6)	320 (7.2)	162 (9.1)	60 (8.4)	20 (10.9)
ARDS at ICU admission	118 (0.6)	94 (2.1)	34 (1.9)	14 (2)	3 (1.6)
Shock at ICU admission	720 (3.8)	337 (7.5)	143 (8)	48 (6.7)	11 (6)
Gastrointestinal bleeding at ICU admission	254 (1.3)	134 (3)	43 (2.4)	17 (2.4)	5 (2.7)
Pneumonia at ICU admission	1506 (7.9)	808 (18.1)	343 (19.3)	144 (20.2)	28 (15.2)
Operations, *n* (%)					
Cardiac surgery	6709 (35.3)	588 (13.1)	141 (7.9)	70 (9.8)	35 (19.0)
Cranial surgery	2730 (14.4)	496 (11.1)	206 (11.6)	76 (10.7)	27 (14.7)
APACHEII scores, median (IQR)	16 (10,20)	20 (15,25)	20 (15,25)	20 (15,24)	19 (16,24)
Intubation sites					
Operating rooms	14,485 (76.3)	1490 (33.3)	617 (34.7)	234 (32.9)	52 (28.3)
Other hospitals	2033 (10.7)	1005 (22.5)	437 (24.6)	189 (26.5)	48 (26.1)
Emergency rooms	1473 (7.8)	1118 (25.0)	398 (22.4)	160 (22.5)	51 (27.7)
Intensive care units	772 (4.1)	492 (11.0)	184 (10.3)	66 (9.3)	12 (6.5)
Tracheostomy	1008 (5.3)	945 (21.1)	687 (38.6)	303 (42.6)	89 (48.4)
Days from the start of ventilation to the onset of the first event onset, median (IQR)	–	–	5 (3, 8)	5 (3, 9)	6 (4, 13)
Outcomes					
Hospital length of stay, median (IQR)	16.00 (12.00, 23.00)	22.00 (15.00, 34.00)	28.00 (17.00, 43.00)	30.00 (19.00, 44.00)	30.00 (21.00, 46.00)
ICU length of stay, median (IQR)	4.00 (3.00, 7.00)	13.00 (8.00, 21.00)	20.00 (12.00, 32.00)	21.00 (14.00, 33.00)	23.00 (15.00, 35.00)
Ventilation days, median (IQR)	2.00 (2.00, 3.00)	8.00 (5.00, 13.00)	14.00 (8.00, 22.00)	15.00 (10.00, 25.00)	20.00 (11.00, 31.00)
Hospital mortality, *n* (%)	1078 (5.7)	614 (13.7)	368 (20.7)	142 (19.9)	41 (22.3)
30-day Hospital mortality, *n* (%)	930 (4.9)	487 (10.9)	262 (14.7)	102 (14.3)	27 (14.7)
ICU mortality, *n* (%)	1005 (5.3)	568 (12.7)	343 (19.3)	129 (18.1)	37 (20.1)
30-day ICU mortality, *n* (%)	993 (5.2)	534 (11.9)	294 (16.5)	113 (15.9)	30 (16.3)
Hospital costs (USD), median (IQR)	11,840.08 (8,251.7, 17,683.14)	18,298.15 (11,263.84, 28,008.99)	25,073.64 (16,050.61, 38,714.54)	27,978.15 (18,653.85, 43,094.25)	33,310.74 (20,275.29, 48,288.4)

*VAE* ventilator-associated events, *ARDS* acute respiratory distress syndrome, *ICU* intensive care units, *GICU* general intensive care units, *SICU* surgical intensive care units, *NICU* neurological intensive care units, *RICU* respiratory intensive care units, *TICU* thoracic surgery intensive care units, *IQR* interquartile range

### The clinical characteristics and pathogen distribution

The characteristics and outcomes of patients with and without VAEs are presented in Table 2. The distribution of age, sex, comorbidities, APACHE II scores, and intubation sites was similar among patients with at least four ventilator-days, regardless of VAEs. Kidney failure was more common among VAE cases (9.1%) than among non-VAE cases with at least four ventilator-days (7.2%), and the proportion was highest among PVAP cases (10.9%). All mechanically ventilated patients without VAE had a greater history of cardiac surgery (35.3%). The most common intubation sites among VAE cases were the operating room (34.7%), followed by other hospitals (24.6%) and emergency rooms (22.4%). There were 184 (10.3%) VAE cases and 492 (11.0%) non-VAE cases intubated in the ICU. Among patients with at least four ventilator-days, 687 (38.6%) and 945 (21.1%) patients with VAE and non-VAE received a tracheostomy, respectively. The median (IQR) time from the start of ventilation to the onset of the first VAC-plus, IVAC-plus, and PVAP was 5 (3–8), 5 (3–9), and 6 (4–13) days.

**Fig. 2:**
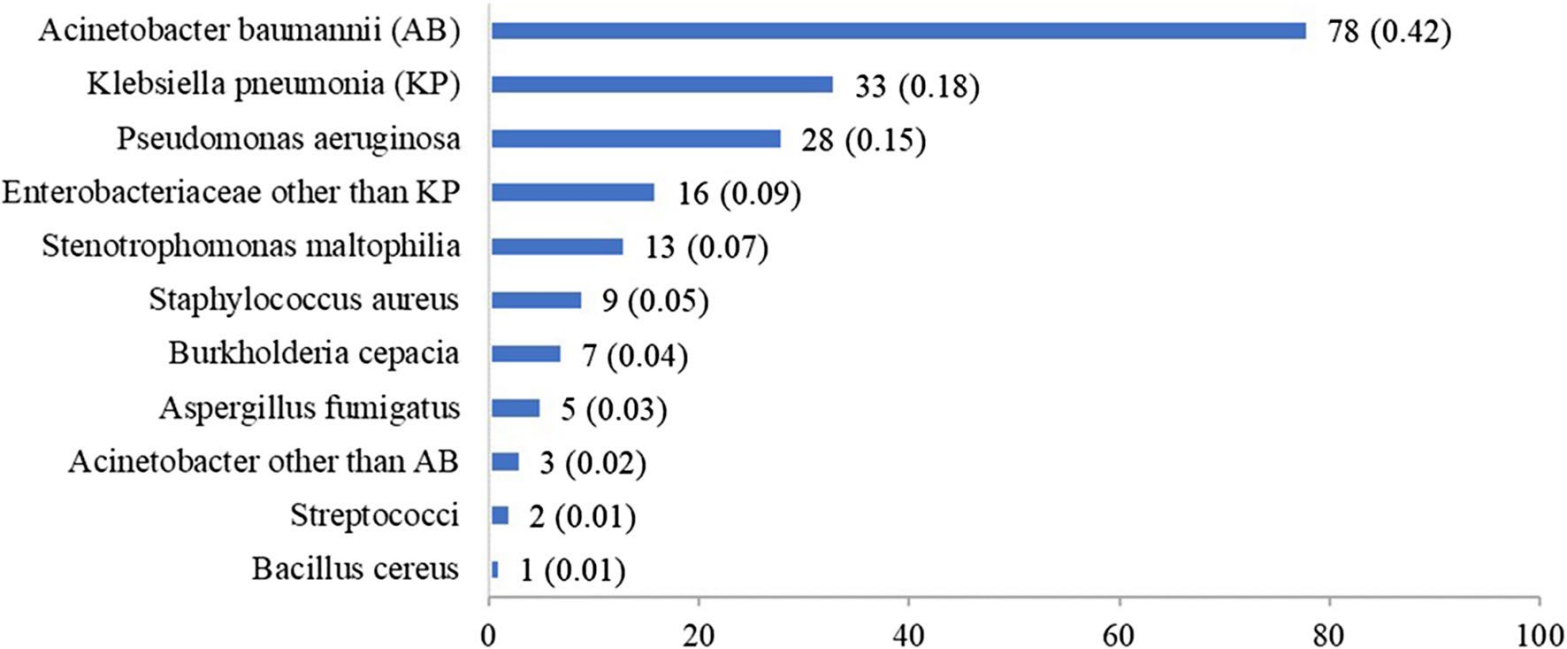
Pathogen distribution in patients with possible ventilator-associated pneumonia

The pathogen distribution in 184 patients with PVAP is presented in Fig. 2. A total of 11 related pathogens were found. The most frequent isolates were *Acinetobacter baumannii* (42.0%), *Klebsiella pneumoniae* (18%), and *Pseudomonas aeruginosa* (15%).

### The compliance rates of processes of care and clinical outcomes of patients

The median (IQR) time of hospital LOS was 28.00 (17.00–43.00), 30.00 (19.00–44.00), and 30.00 (21.00–46.00) days for patients with VAC-plus, IVAC-plus, and PVAP, respectively, which were all longer than for those without VAEs. The ICU LOS and ventilation duration were also longer among patients with VAEs (20 [12, 32] vs. 13 [8, 21]). A total of 368 patients with VAEs died during hospitalization, with 343 deaths occurring in the ICUs, corresponding with higher hospital mortality (20.7%) than that seen in patients without VAEs (614 [13.7%]). The total hospitalization costs for VAEs were more than twice those for non-VAE patients with at least one ventilator-day (25,073.64 USD vs. 11,840.08 USD), and nearly 1.5 times those for non-VAE with at least four ventilator-days (25,073.64 USD vs. 18,298.15 USD).

The compliance rate of head-of-bed elevation, oral care, and paired spontaneous awakening trials and breathing trials was 99.0%, 98.8%, and 98.7%, respectively. The compliance of stress ulcer prophylaxis and thromboembolism prophylaxis was not mandated in ICUs with a 59.6% and 77.8% compliance rate, respectively (Table 3).

**Table 3:** The compliance rates of processes of care

	Total EMV (N = 21,723)
Process of care, *n* (%)	
Head-of-bed elevation	21,507 (99.0)
Oral care	21,456 (98.8)
Paired spontaneous awakening trials and breathing trials	21,442 (98.7)
Stress ulcer prophylaxis	12,952 (59.6)
Thromboembolism prophylaxis	16,890 (77.8)

*EMV* episodes of mechanical ventilation

## Discussion

To our knowledge, this is the first study to describe detailed epidemiological data of VAE based on a routinely active surveillance system in China. In this study, we found that the majority of patients admitted to ICUs required MV. Among all patients on MV, the rate of VAEs was relatively low, in that only 8.6% met the criteria for VAE (16.7 per 1000 ventilator-days) and less than 1% met the PVAP criteria (1.64 per 1000 ventilator-days). However, among patients with at least four consecutive ventilator-days, the rates reached 28.5% for VAC-plus, 11.4% for IVAC-plus, and 2.9% for PVAP, respectively. The rates of all three VAE tiers varied in different ICU units and were highest in the NICU. A total of 1616 (85.9%) VAEs occurred within 14 days after receiving MV with the highest mean rate within 7 days at 5.92 per 100 EMV and 18.65 per 1000 ventilator-days. The most common pathogen in patients with PVAP was Acinetobacter *baumannii*, which accounted for almost half of all isolates. All three tiers of VAEs were associated with longer duration of MV, prolonged ICU and hospital stays, increased hospital and ICU mortality, and higher costs than in patients without VAE, especially in the PVAP group.

The VAE rates varied significantly in previous studies, mainly because the denominator used to calculate VAE rate differed among individual studies. For instance, some studies only recruited patients with at least 48 h (7–40.8 per 1000 ventilator-days) [1, 2, 19, 25, 27, 30, 31, 38], 4 days (6–13.8 per 1000 ventilator-days) [21, 22, 24, 27, 35] or 5 days (107 per 1000 ventilator-days) [4] on a ventilator, while some studies included all mechanically ventilated patients (6.3–14.4 per 1000 ventilator-days) [7, 17, 23, 26, 28, 33, 34, 36]. The rates of VAC-plus in our study were slightly higher than in studies with the same VAE definition and all MV episodes as the denominator. This is likely because VAE cases were prospectively and actively identified in our study. The values of PEEP and FiO2 were recorded on an hourly basis. A threshold-based warning system derived from these records was implemented. Once PEEP or FiO2 reached the threshold, an alarm would be triggered, and infection control practitioners would immediately check the patient. The system provided a useful approach to identify all potential cases, leading to a higher rate than that of other retrospective studies. Moreover, as a national critical care center in Western China, ICU patients at WCH had relatively serious illnesses and thus may be more prone to developing VAE. The rates in studies that restricted eligibility to patients with at least 2 ventilator-days were almost higher than 20 per 1000 ventilator-days [4, 19, 25]. In addition, differences in rates depended on ICU type as shown in our study and previous studies [17]. We found that the VAC-plus rates were 23.7, 21.4, and 7.3 per 1000 ventilator-days in the SICU, NICU, and TICU, respectively, whereas Klomps et al. reported 16.0, 9.8, and 12.9 per 1000 ventilator-days in these three units, respectively [17]. Zhu and Magill et al. also found that the VAE rates of the major teaching hospitals were much higher than those in non-major teaching municipal hospitals [7, 28]. In the three tiers of VAE definition, PVAP is a closer proxy for traditional VAP, and rates were 0.9 per EMV and 2.2 per 1000 ventilator-days in our cohort, which was similar to some previous studies [7, 17, 21, 38].

Most patients may be more prone to VAEs early in the ventilation episode. The median time from the start of ventilation to the first event onset in this study was 5 days for both the VAC-plus and IVAC-plus groups, while it was 6 days for the PVAP group, consistent with other reports [1, 2, 4, 17, 19]. In this study, most VAEs occurred within the first two weeks from the initiation of MV. The mean rate in the first week was more than three times that in the second week and dropped sharply to less than one per 100 patients after 14 days. Similar trends were shown in the study by Klomps et al., wherein patients were more prone to VAEs early in the course of MV as a consequence of the acute interventions performed to stabilize the patient’s presenting illness [17].

Due to the poor correlation between VAE and the traditional definition of VAP, the new approach has not been widely implemented outside the USA and is mainly used for surveillance [16]. However, VAE is not designed to be a proxy for VAP, rather it was intentionally to broaden the surveillance from targeting pneumonia only to include the complications associated with ventilation in healthcare settings [13]. Therefore, many VAEs may be conditions other than pneumonia. Nevertheless, surveillance aims to identify patients with severe complications and to track the impact of prevention strategies. With shifting the focus on all major causes of respiratory deterioration in ventilated patients, VAE surveillance imposes a severity threshold to identify a subset of patients with severe disease. Indeed, previous studies have found that patients without impaired gas exchange are associated with a relatively benign clinical course [39]. Compared to those with traditionally defined VAP, patients with VAE had a higher mortality rate as demonstrated by our study group [7] and that others [2, 40]. This confirms the clinical importance of VAE surveillance. In addition, traditional ventilator bundles were primarily based on subjectively and nonspecifically traditional VAP definition, but some components proved to be harmful [41–47]. VAE surveillance, which is defined based on objective ventilator data, is less vulnerable to misattributing benefit to neutral or negative interventions [13]. Several potential strategies for preventing VAEs have been proposed including minimizing sedation, paired daily spontaneous awakening and breathing trials, and conservative fluid management [44]. Studies have suggested that the VAE prevention bundles were associated not only with lower risks of VAE, but also with less time to extubation and shorter LOS in hospital [2, 45, 48–51]. In general, VAE is a relatively new algorithm to address complications associated with ventilation including, but not restricted to, VAP. This is quite different from the traditional approach to address VAP alone and therefore brings challenges for the long-standing thinking pattern and practice routines in patient care. It may take time for such a relatively new algorithm to be adopted widely in clinical practice. More studies are warranted to further demonstrate the preventability of VAE and its clinical significance on patient outcomes in different countries and among different patient populations. These studies will be likely to generate more high-quality, convincing, and patient care-focused (in addition to the surveillance purpose) evidence to further demonstrate the clinical relevance of VAE. Meanwhile, as VAE is simple and can be easily applied, we recommend clinicians to raise awareness towards VAE and to consider performing observations of the incidence and impact of VAE for their patients.

This study was based on a routinely prospective surveillance system, which is so far unique in China. Compared with retrospective investigations, it is less likely to miss VAE cases, and the diagnosis of VAE has been validated as relatively accurate. However, our study has some limitations. Our findings were based on data from one tertiary hospital, which may not be generalizable to other settings. The preferential practices may vary remarkably in ICU patients with MV across hospitals with different levels or in different countries. Second, as we did not monitor traditional VAP, we were unable to compare the differences between traditional VAP and PVAP.

## Conclusion

The majority of ICU patients required MV. VAEs were common among those with ≥ 4 ventilator-days and occurred early in the course of MV. All tiers of VAE were highly correlated with poor clinical outcomes, including longer stays in the hospital and ICU and an increased risk of mortality. These findings suggest the importance of VAE surveillance and of developing strategies to prevent VAEs. However, uniformed inclusion criteria and patient populations used as the denominator for calculation of VAE rate should be carefully addressed in future studies.

## Data Availability

The datasets used and/or analyzed during the current study are available from the corresponding author on reasonable request.

## Abbreviations

VAP: Ventilator-associated pneumonia
HAI: Hospital-acquired infection
ICU: Intensive care unit
VAE: Ventilator-associated event
MV: Mechanical ventilation
PVAP: Possible ventilator-associated pneumonia
VAC: Ventilator-associated condition
IVAC: Infection-related ventilator-associated complication
CDC: The Centers for Disease Control and Prevention
WCH: West China Hospital
EMR: Electronic medical record
GICU: General intensive care unit
SICU: Surgical intensive care unit
NICU: Neurological intensive care unit
RICU: Respiratory intensive care unit
TICU: Thoracic surgery intensive care unit
PICU: Pediatric intensive care unit
NHSN: National Healthcare Safety Network
PEEP: Positive end-expiratory pressure
FiO2: Fraction of inspired oxygen
ARDS: Acute respiratory distress syndrome
ICD-10: International Classification of Diseases, 10th edition
IQR: Interquartile range
LOS: Length of hospital stay
EMV: Episodes of mechanical ventilation
